# A modified oblique incision in hamstring tendon graft harvesting during ACL reconstruction

**DOI:** 10.1186/s13018-021-02341-5

**Published:** 2021-03-22

**Authors:** Biao Zhu, Xuelei Li, Tengteng Lou

**Affiliations:** 1 Department of Joint Orthopaedic Surgery and Sports Medicine, Xuzhou Medical University Affiliated Hospital of Tengzhou Central People’s Hospital, Xingtan Road 181, Tengzhou, 277500 Shandong China; 2 Department of Orthopedics, Guanxian People’s Hospital, Dongfeng West Road 51, Liaocheng, Guanxian, 25250 Shandong China; 3 Postpartum Health Care Department, Maternal and Child Health Hospital of Tengzhou, Longquan Road 3966, Tengzhou, 277500 Shandong China

**Keywords:** Anterior cruciate ligament, Inferior patellar branch of saphenous nerve, Hamstring tendon

## Abstract

**Background:**

During anterior cruciate ligament (ACL) reconstruction, different methods of harvesting hamstring tendon may lead to different degrees of injury to the inferior patellar branch of the saphenous nerve (IPBSN). Most of recent studies in the literature suggest that the classic oblique incision (COI) can reduce the incidence of IPBSN injury. We proposed a modified oblique incision (MOI) and compared it with the COI in terms of the resulting levels of injury and sensory loss and the clinical outcome.

**Methods:**

Patients with ACL injury admitted to our hospital from April 2015 to July 2019 were randomly selected and included in our study. Thirty patients underwent the COI to harvest hamstring tendons, and the other 32 patients underwent the MOI. The pin prick test was performed to detect the sensation loss at 2 weeks, 6 months, and 1 year after the operation. Digital photos of the region of hypoesthesia area were taken, and then, a computer software (Adobe Photoshop CS6, 13.0.1) was used to calculate the area of the hypoesthesia. The length of the incision and knee joint functional score were also recorded.

**Results:**

At the final follow-up, the incidence of IPBSN injury in COI and MOI were 33.3% and 9.4%, and the areas of paresthesia were 26.4±2.4 cm^2^ and 9.8±3.4 cm^2^ respectively. There was no significant difference in the incision length or knee functional score between the two groups.

**Conclusion:**

The MOI can significantly reduce the risk of injury to the IPBSN, reduce the area of hypoesthesia, and lead to high subjective satisfaction. Therefore, compared with the COI, the MOI is a better method of harvesting hamstring tendons in ACL reconstruction.

## Introduction

Arthroscopic ACL reconstruction can effectively stabilize the knee joint and restore function. It is a well-established and satisfactory surgical technique for the treatment of ACL rupture. The hamstring tendon is an important source of autologous tendon grafts at present, and hamstring tendon harvesting is more convenient than are other methods and can achieve the same effect [7]. However, hamstring tendon harvesting may cause saphenous nerve injury, especially to the inferior patellar branch, which can lead to anterior tibial hypoesthesia, neuropathic pain, and painful neuroma [31]. The incision that is made for hamstring tendon harvesting is close to the IPBSN, and the exact location of the incision is highly variable. Therefore, the position and direction of the incision made for hamstring tendon harvesting are very important factors that can be modified to the occurrence of IPBSN injury [4].

The saphenous nerve innervates the sensory nerves in the skin around the knee joint, leg, and ankle [2]. It originates from the posterior part of the femoral nerve in the proximal femur and enters the adductor canal on the medial side. After leaving the adductor canal, it divides into two terminal branches: the infrapatellar branch and the sartorius branch. The IPBSN then divides into two more branches, the upper and lower branches, which innervated the anteromedial sensory nerves in the skin of the knee. The location of the IPBSN varies across individuals and between the two lower limbs of the same individual. Thus, hamstring tendons are very vulnerable to injury in the pes anserinus. It has been reported that the IPBSN can be damaged by a minimally invasive incision and blunt contusion by a tendon-harvesting device [29].

In recent years, many studies have recommended against performing a standard incision for harvesting hamstring tendons. Most of many studies have confirmed that oblique incision can significantly reduce the risk of injury to the IPBSN compared with vertical and transverse incision [12, 20, 24, 27, 32]. From these studies, we identified two types of oblique incisions. One type is the oblique incision proposed by Brown et al. [4] which is parallel to the upper edge of the pes anserinus and Langer’s line. The other type of oblique incision is centered at and located three fingerbreadths below the joint line, 1-cm medial to the tibial tubercle, and 1- to 3-cm distal to the tubercle [14]. In fact, the positions described by the two incisions are roughly the same. We named it the classical oblique incision (COI). We also found that, in these studies, the IPBSN injury rates varied largely from 12 to 86%. These complications can affect patient satisfaction with surgery. Therefore, we searched references and found that Kerver et al. [17] studied the anatomy of the IPBSN and proposed the concept of a safe area for it; this same region is similar to the oblique incision position described by Boon et al. [3]. We then discovered that the COI is not in this identified safe area, especially when the incision is long. Therefore, we designed a new and more accurate oblique incision that is located within this safe area. We named it the modified oblique incision (MOI). At present, there are no clinical studies on the design of incisions based on this safety zone. The purpose of this study was to compare the postoperative complications of two kinds of oblique incisions and confirm whether this MOI can reduce complications to a greater extent than can the COI and yield satisfactory clinical effects.

## Materials and methods

From April 2015 to July 2019, 62 patients with ACL tears were hospitalized in our hospital. The exclusion criteria were as follows: an injury spanning multiple ligaments, a meniscus tear with sutures, a history of injury and surgery of the knee joint, and nervous system diseases. The inclusion criteria were as follows: a simple ACL rupture or meniscus injury treated only with meniscus plasty. There were 54 males and 8 females aged 19 to 48 years. In this study, the COI was performed in 30 patients, and the MOI was performed in 32 patients. The average age of the patients was 30.2 years old (31.3 years old with the COI and 29.5 years old with the MOI), and there were 37 cases in the right knee joint (Table 1). Informed consent was obtained from the patients prior to the study. The study was approved by the medical ethics committee of our hospital.

**Table 1:** Demographic values of the patients

	COI	MOI	Total	*P* value
Sex (male/female)	26/4	28/4	62/8	>0.05
Age (mean ± SD)	31.3±3.2	29.5±4.3	30.2 ±4.5	>0.05
Side right (%)	18 (60%)	19 (59%)	37 (60%)	>0.05

*SD* standard deviation, *COI* classic oblique incision, *MOI* modified oblique incision

**Fig. 1:**
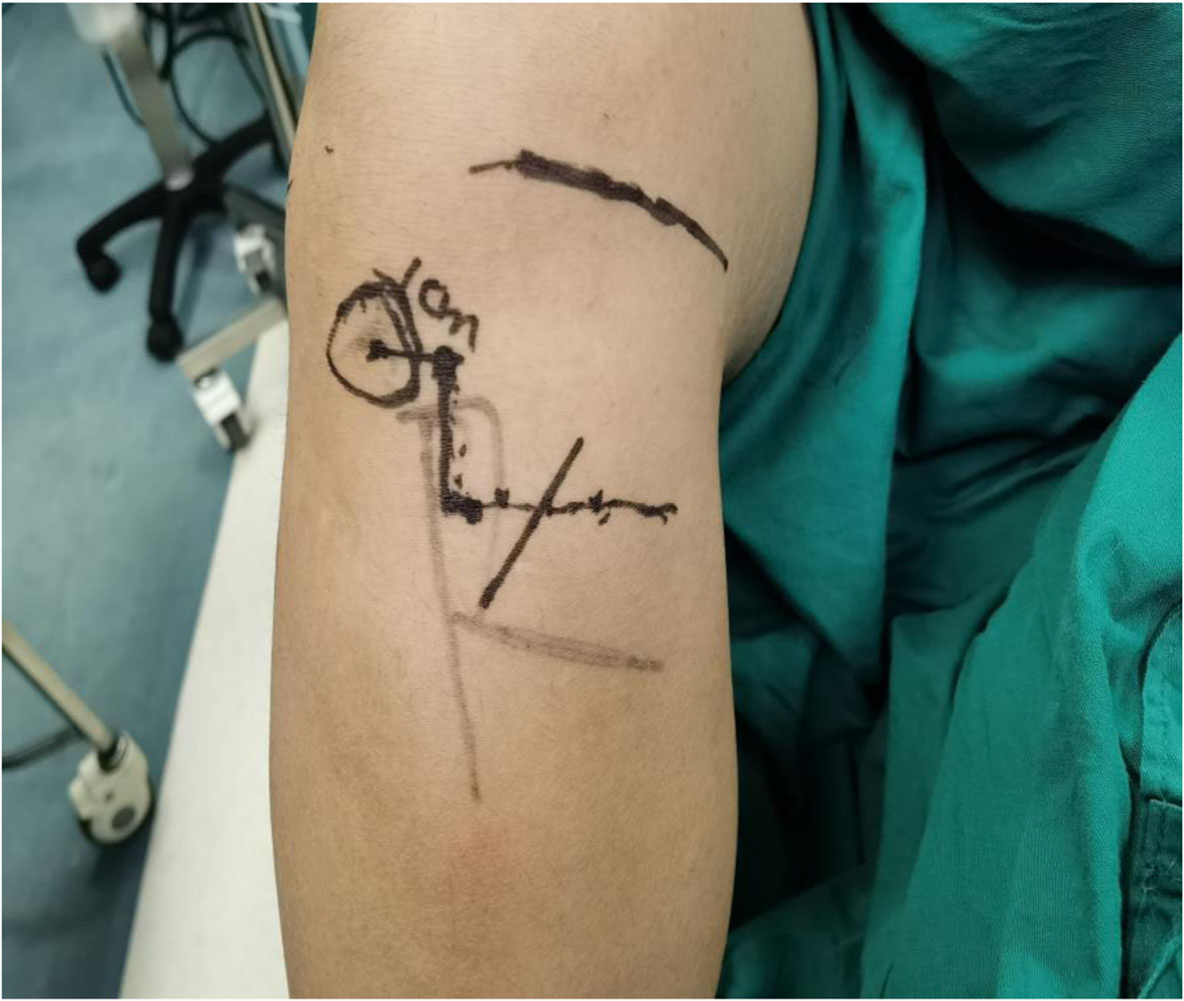
COI—the incision is centered at and located three fingerbreadths below the joint line, 1-cm medial to the tibial tubercle, and 1- to 3-cm distal to the tubercle

**Fig. 2:**
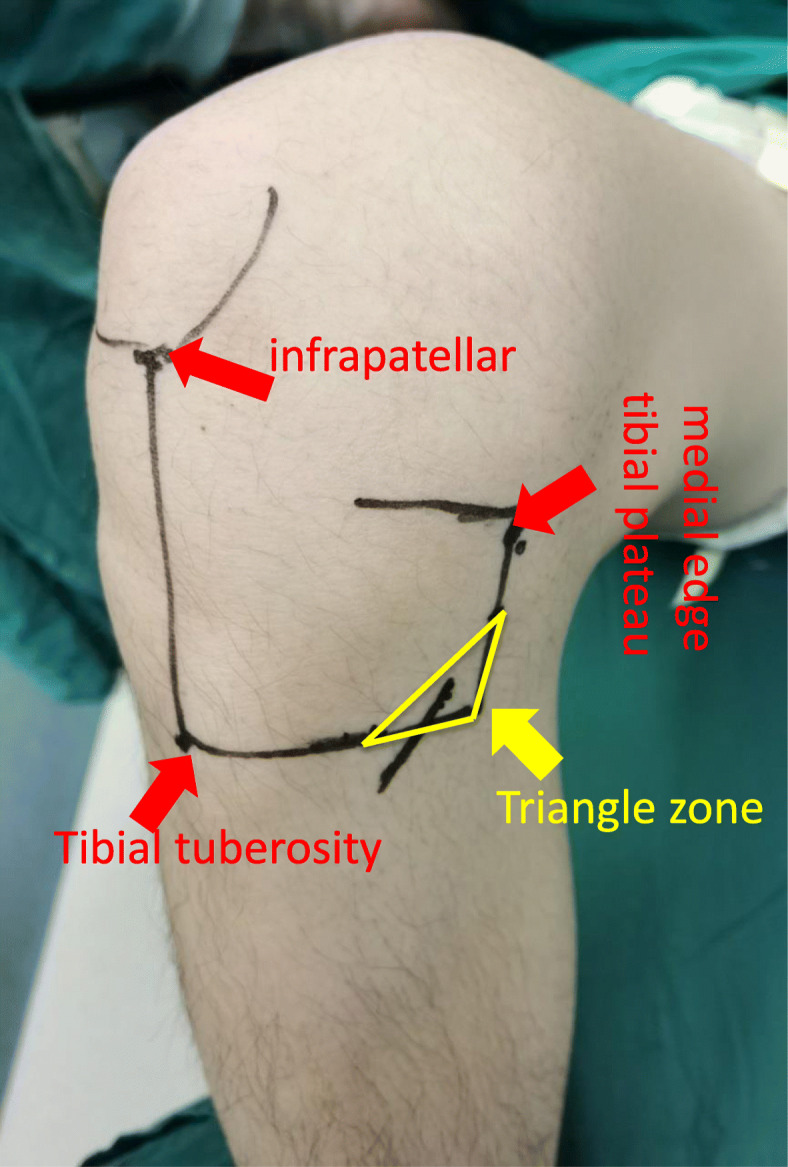
Triangle zone—the medial and inferior low risk zone of the IPBSN is a triangle that a vertical line is projected downward from the medial edge of the tibial plateau and a horizontal line from the tibial tuberosity to the medial side of the knee. The zone is located distally from 50% of the vertical line and medially from 66% of the horizontal line. MOI—the incision midpoint was median point of the bottom edge of the triangle, through this midpoint and the oblique angle with the bottom edge was 51° and extended posteriorly and anteriorly

All patients were diagnosed with ACL tears by a clinical physical examination and MRI, and then, ACL reconstruction was performed. First, an arthroscopic examination was performed to further confirm the ACL was ruptured, and then, the hamstring tendon was harvested. After the semitendinosus tendon and gracilis tendon were palpated by hand, the incision position was determined. The COI proposed by Kalthur et al. [14] is made three fingers below the knee joint line, 1-cm medial to the tibial tubercle, and 1- to 3-cm distal to the tubercle, and above the semitendinosus tendon and gracilis tendon, the oblique incision is made along the skin Langer’s line (Fig. 1). According to the medial and inferior triangle safe area of the IPBSN proposed by Boon et al. [3] and Kerver et al. [17] who described the triangle as a zone located distally from 50% of the vertical line which is projected downward from the medial edge of the tibial plateau and medially from 66% of the horizontal line that from the tibial tuberosity to the medial side of the knee, we designed the MOI so that the median point of the bottom edge of the triangle was the incision midpoint, and the oblique angle with the bottom edge was 51° and extended posteriorly and anteriorly (Fig. 2). The length of the incision was extended as needed. After the skin incision was made, a blunt dissection technique was used for subcutaneous fatty tissue with vascular forceps to reduce the risk of injury to the IPBSN and excessive pulling of the incision when the hamstring tendon was separated and exposed. Similarly, the subcutaneous fatty tissue was carefully sutured to reduce the risk of injury to the IPBSN. In both methods, the knee joint was flexed to 90°, and the hip joint was externally rotated because this posture can reduce the risk of saphenous nerve injury during tendon harvesting [2].

ACL reconstruction was performed by the single-bundle anatomic technique using standard arthroscopy instruments. The medial-superior portal was used to observe the approach, and the anteromedial inferior portal was used to locate and reach the femoral tunnel. The tibial tunnel was cut using the same graft incision with the guide size adjusted to 55. The doubled fourfold hamstring tendon graft was fixed to the femur with the Endo Button (Smith and nephew), and the tibial side was fixed with an absorbable compression screw with a sheath. The length of the skin incision was measured after suturing. A consistent rehabilitation schedule was enforced postoperatively. Isometric contraction training of the quadriceps femoris was started on the second day after the operation. On the third day, partial weight-bearing activities were carried out on crutches under the protection of a brace. Passive flexion of knee joint to 90° and complete extension were reached within 2 weeks after the operation. From 6 to 8 weeks after the operation, the flexion angle reached 120°.

**Fig. 3:**
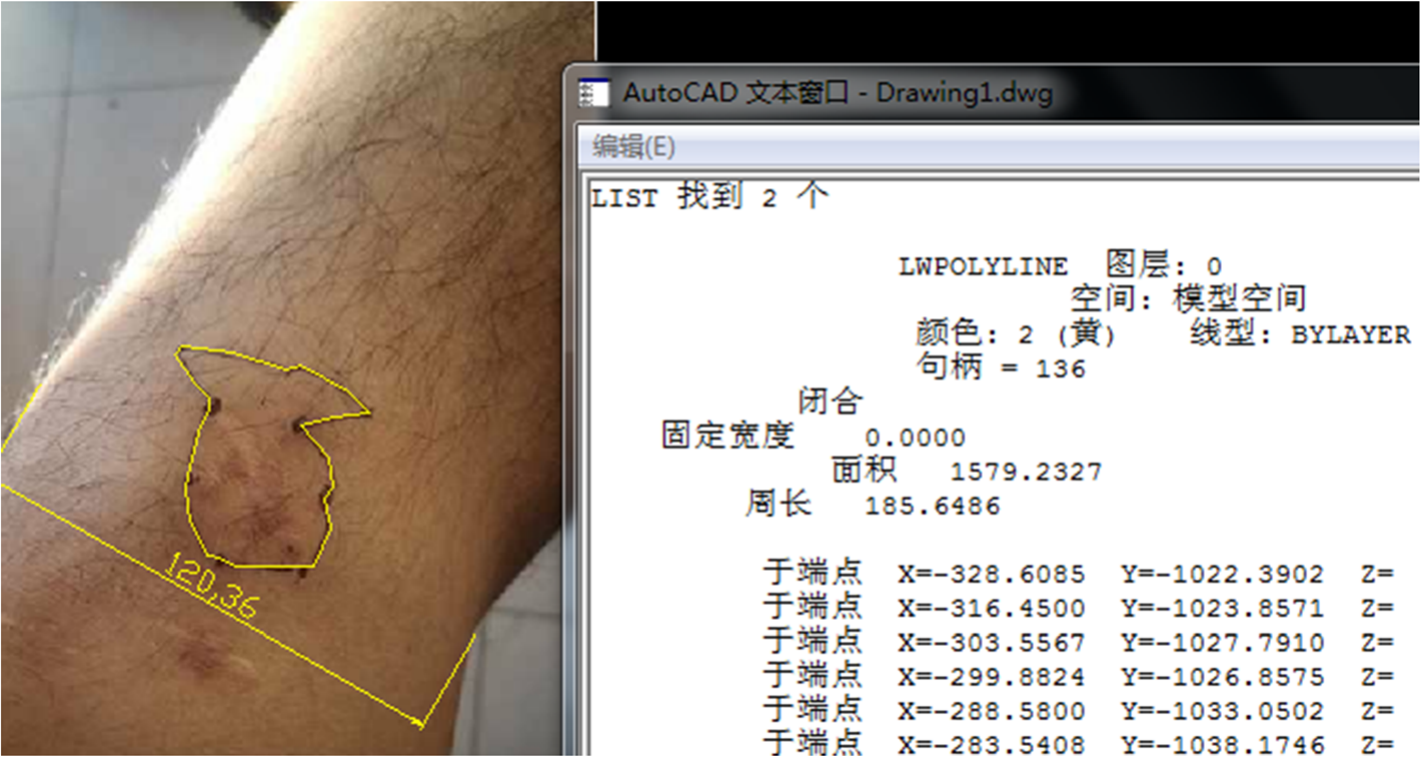
The paresthesia area of a patient at 1-year follow-up was measured by a computer software (Adobe Photoshop CS6, 13.0.1)

All patients were examined for skin paresthesia at 2 weeks and 1 year after the operation. For the examination, a blunt needle was used to prick the skin from the proximal end of the incision, the points ranging from abnormal sensation to normal sensation were marked, and finally, these points were connected. Then, a camera was used to take pictures, and the area of sensory abnormalities was measured using a computer software (Adobe Photoshop CS6, 13.0.1) (Fig. 3). At the final clinical follow-up, the Lysholm score and International Knee Documentation Committee Subjective Knee (IKDC) assessment form [13] were assessed to evaluate the joint function.

The differences in incision length and demographic data between the COI group and the MOI group were compared by two independent sample *t*-test. *F* analysis was used for the comparison of the paresthesia areas, and *P* values of <0.05 were considered statistically significant. A priori statistical power analysis was performed with the percentage of persistent sensory loss at 1 year as the primary outcome variable, and *P* < 0.05 indicated a statistically significant difference. The SPSS software (SPSS for Windows version 12) was used for statistical analysis.

## Results

Two weeks after the operation, 12 (40.0%) patients in the COI group and 6 (15.6%) patients in the MOI group exhibited a loss of sensation in the anterior knee area. At the final follow-up, 10 (33.3%) of the patients showed hypoesthesia in the COI group and 2 (9.4%) of the patients in the MOI; the difference was significant. No patients had posterior thigh pain, neuropathic pain, or painful neuroma. The average incision length of the COI was 3.1 cm ± 0.87 cm (1.8–4.8 cm), and the average length of the MOI was 2.9 cm ± 0.85 cm (2.1–4.2 cm), and the difference between the two groups was not significant. The paresthesia area for the COI and MOI groups were 42.1±3.5 cm^2^ and 17.3±2.6 cm^2^ at 2 weeks follow-up and 26.4±2.4 cm^2^ and 9.8±3.4 cm^2^ at the 1-year follow up, respectively. The difference was statistically significant. At the final follow-up, the Lysholm knee score and IKDC subjective assessment of the two groups markedly improved in both groups, and there was no significant difference between the two incision groups (Table 2).

**Table 2:** Outcomes of incision surgery by group

	COI *N*=30	MOI *N*=32	*P* value
Number of patients with numbness			
2nd week (%)	40.0%	15.6%	< 0.05
1 year (%)	33.3%	9.4%	< 0.05
Area of sensory loss (mean + SD) cm^2^			
2nd week (%)	42.1 ± 3.5	17.3 ± 2.6	< 0.05
1 year (%)	26.4 ± 2.4	9.8 ± 3.4	< 0.05
Incision length (mean ± SD) cm	3.1±0.87	2.9 ± 0.85	> 0.05
Lysholm knee score 1 year (%)	93	94	>0.05
IKDC subjective assessment 1 year (%)	95	97	>0.05

## Discussion

IPBSN injury is a common potential complication of operations performed around the knee joint [22]. Because the saphenous nerve and its branches are very close to the incision, and the IPBSN location varies widely cross individuals, even with minimally invasive incision, the saphenous nerve and its branches are still at risk of injury. In recent years, the risk ratio of IPBSN in ACL reconstruction has been reported to be 12–84% [8, 16, 18, 31]. These injuries are mainly related to the harvesting of autologous tendon grafts. In some studies, the authors recommend using an autologous hamstring tendon graft to replace part of the patellar tendon because hamstring tendon harvesting can reduce the risk of other complications to a greater extent than can patellar tendon harvesting, and the procedure is relatively simple [10, 16, 21]. However, hamstring tendon harvesting can increase the incidence of IPBSN injury [6].

The saphenous nerve is the longest branch of the femoral nerve and the longest nerve branch relevant to human walking [24]. After this nerve exits the adductor canal, it passes behind the sartorius muscle and travels along the surface of the gracilis tendon on the postero-medial side of the joint line, and then, it divides into the lower patellar branch and the sartorius branch [6, 11]. This anatomical structure is very close to the hamstring tendon, so this nerve is easily injured when the hamstring tendon is harvested [3]. The sartorial branch of the saphenous nerve extends along the medial part of the tibia toward the distal end of the superficial peroneal nerve on the dorsal side of the second metatarsal bone. The saphenous nerve has only sensory nerve fibers, which innervate the superficial sensation of the medial knee, anterior patella (saphenous nerve, patellar lower limb), medial tibial crest, posterior medial leg, and medial foot [1]. It has been reported that injury to the inferior patellar branch leads to the loss of superficial skin sensation, especially in the anterior and medial areas of the patella. Therefore, some patients have difficulty kneeling and walking due to knee-related issues [15].

The incidence of IPBSN injury has a direct and very important relationship with the type of incision selected for hamstring tendon harvesting [28]. It has been reported that the IPBSN is closely related to the location, type, and direction of the incision [17]. In recent years, most studies have reported that oblique incisions are less likely to damage the IPBSN than are vertical and horizontal incisions because according to autopsy studies, oblique incisions are more parallel and farther away from the IPBSN, so they are located in a safer area [20, 26–30, 32]. However, horizontal incisions easily lead to injury of the sartorius branch [24, 25].

Haviv et al. [10] reported that the injury rate related to transverse incision was 43% and that related to vertical incisions was 59%. Papastergiou et al. [24] reported an IPBSN injury rate of 14.9% with transverse incision and 37.9% with vertical incisions. A few authors also reported that there are no differences between the two kinds of incisions and that the injury risk rate is as high as 88% [18]. In recent studies, the risk of IPBSN injury was lower for oblique incisions than for two other types of incisions [9, 28]. Henry et al. [12] reported that the risk of injury with vertical incisions was 64.7%, that with transverse incisions was 50.0%, and that with oblique incisions was 27.6%. Pękala et al. [26] reported that the risk of injury with vertical incisions was 51.4%, that with oblique incisions was 26%, and that with transverse incisions was 22.4%. All the oblique incisions performed by the authors were COIs, but the reported rates of IPBSN are quite different. According to a study on the anatomy of the IPBSN by Kerver et al. [17], a safe area for the IPBSN has been proposed. We found that part of the COI was not located within the safe area, especially when the incision was long. This finding may explain why the rate of IPBSN injury for the COI differs from that for the oblique incision. However, in my research, the rate of IPBNS injury was 33.3% in the COI group and 9.4% in the MOI group at the final follow-up, and the areas of numbness were 26.4±2.4 cm^2^ and 9.8±3.4 cm^2^, respectively. Regarding the rate of IPBSN injury or the area of hypesthesia, there was significant difference between groups. Moreover, the type of incision that we proposed is more accurate and easier to perform than that is the COI. Therefore, we think it is a more appropriate method of harvesting hamstring tendons.

According to the literature, blunt injury by a tendon-harvesting device is also a cause of saphenous nerve injury [23]. However, it has been reported that there are fewer nerve injuries associated with tendon harvesting than there are nerve injuries associated with incisions [2, 29]. Sanders et al. [29] considered that the tendon-harvesting device may only damage the suture branch of the saphenous nerve, while an incision in the hamstring tendon may damage the IPBSN. Almost all the authors of previous studies believe that it is possible to avoid saphenous nerve injury caused by the tendon-harvesting device by keeping the knee flexed and thigh rotated during hamstring tendon harvesting because the saphenous nerve moves backward and is located far away from the incision [6, 23]. In this study, two kinds of oblique incisions were made in this position to harvest hamstring tendons. In our study results, none of the patients experienced injury to the suture branch, which can cause an abnormal sensation in the medial and distal tibial ridge, as well as sensory abnormalities in the anteromedial skin of the knee joint.

Some authors believe that the length of incision may affect the risk of injury of the IPBSN [18]. Henry et al. [12] conducted an autopsy study and found that in patients who did not experience an IPBSN injury, the distance between various incisions and the IPBSN was very small, with an average distance of 8.2–8.7 mm. Therefore, he believed that the length and direction of the incision were important, and that the length of the incision should be minimized. This also explains why the probability of IPBSN injury was reported to be relatively high (as high as 84%) in the study by Kjaergaard et al. [18], even when an oblique incision was performed. We also found that the COI is not contained completely within the safe area that was proposed by Kerver et al. [17] and Boon et al. [3], especially when the incision is long. Therefore, the risk of injury to the IPBSN is high. In our study, the average incision lengths for the COI and MOI methods were 3.1 cm ± 0.87 cm and 2.9 cm ± 0.85 cm, respectively, with no significant difference, which reduced the risk of saphenous nerve injury caused by a long incision.

However, a shorter incision makes it difficult to expose the tendon. To expose the tendon, an excessive stretching incision may lead to blunt nerve traction injury. Some authors suggest that surgeons used the blunt separation technique to avoid overstretching and close the wound carefully. This method can also reduce the risk of injury to the IPBSN [12]. In this study, in patients whose level of sensation returned to normal within 3–6 months after surgery, the recovery of sensation was considered to be related to avoid overstretching and close the wound carefully. This study was completed by a single surgeon, and thus, inter-surgeon variability can be excluded. The average length of the incisions was 3.1 cm ± 0.87 cm for the COI and 2.9 cm ± 0.85 cm for the MOI in this paper. We found that all 6 patients with skin sensory loss were obese patients with sensory loss in the lower limbs. The causes of skin sensory loss may be due to the surface markers being placed incorrectly due to difficulty palpating the locations on the body, excessive stretching, and a relatively long incision for tendon exposure.

Although Kerver et al. [17] and Boon et al. [3] proposed a safe area, the variation in the location of the IPBSN is high. They proposed the concept of a safe area on the basis of a limited number of autopsy studies, which is also a limitation of their work. Therefore, we think that the saphenous nerve injury in patients who undergo the MOI may be related to variations in the location of the saphenous nerve.

In recent years, most of the related studies that have been conducted have reported that regardless of the type incision that was selected, there were no statistically significant differences in postoperative functional scores. Most patients with abnormal sensation around the knee thought that their work and life would not be affected [20, 26–30, 32]. In this study, there was no significant difference in knee joint function or subjective feeling between the two incision groups.

Posterior thigh pain is another complication after hamstring autograft harvesting. Laakso et al. [19] reported that ten athletes (7 males and 3 females) had experienced an injury to the harvested hamstring site after an ACL reconstruction and underwent operative treatment and obtained good result. They demonstrated that an early trauma during rehabilitation could commence more retraction to the harvested muscle itself and affect the regeneration of the neotendon. In our study, we found no posterior thigh pain at final follow-up. This may be because none of our patients are athletes.

MOI incision can easily recognize and expose the accessory tendon insertions for hamstring tendon and reduce the incidence of tendon amputation. In a review study, Charalambous and Kwaees [5] overview the hamstring tendon anatomy and found that the reason of premature tendon amputation and short graft is accessory tendon insertions and fascial bands which site proximal to the insertion of pes anserinus. Our incision is closer to the proximal tendon than COI. So MOI incision is easier to expose the accessory tendon insertions and fascial bands and reduce the incidence of premature tendon amputation and short graft.

This study had some limitations. The first limitation is that observer bias may have affected the results. All the data were collected and measured by a staff member. Due to the busy working conditions in our general hospital, no other observers could assist with the study. Second, the sample size is small, limiting the generalizability of the results, and studies with larger sample sizes need to be conducted to confirm the results. Moreover, when the skin incision position was located, we relied on the palpable anatomical structures on the body surface, so there may have been errors in locating the position for each incision, thereby affecting the results. The third is that due to the small sample size, the results may be biased. Finally, blunt needle acupuncture is not accurate enough to be used to measure the extent of sensory nerve injury. In the future, electrophysiological studies may be used to assess nerve injury more accurately.

All the previous studies have demonstrated that the IPBSN always exists regardless of the type of surgical incision selected and that the incision needs to be performed carefully. Although the MOI performed in this study can significantly reduce the risk of injury, injuries cannot be completely avoided. Mild paresthesia will not affect a patient’s life or knee joint function. The area of hypesthesia gradually decreases with time and even recovers completely. Therefore, the risk of postoperative nerve injury should be explained to patients before surgery, but generally, this injury will not affect function.

## Conclusions

Compared with the COI, the MOI is associated with a lower risk of injury to the IPBSN, and the area of sensory loss is also smaller. Therefore, the MOI can be considered a better alternative for harvesting hamstring tendons in ACL reconstruction.

## Data Availability

The datasets used and/or analyzed during the current study are available from the corresponding author on reasonable request.

## Abbreviations

ACL: Anterior cruciate ligament
IPBSN: Inferior patellar branch of the saphenous nerve
COI: Classic oblique incision
MOI: Modified oblique incision
IKDC: International Knee Documentation Committee Subjective Knee
